# Differential Expression of Interleukin-6 (IL-6), Interleukin-10 (IL-10), and Interleukin-1 Beta (IL-1β) in Insulin Resistance and Type 2 Diabetes: A Comparative Study of Messenger RNA (mRNA) and Protein Levels

**DOI:** 10.30699/ijp.2025.2056657.3433

**Published:** 2025-08-15

**Authors:** Hawraa Fadhel Gaber, Muslim Idan Mohsin

**Affiliations:** Pathological Analysis Department, Faculty of science, University of Kufa, Kufa, Iraq

**Keywords:** interleukin-6, interleukin-10, interleukin-1 beta, diabetes mellitus, insulin resistance, type 2 diabetes mellitus

## Abstract

**Background & Objective:**

This study aimed to elucidate the complex interplay between insulin resistance (IR), type 2 diabetes mellitus (T2DM), and the expression of key inflammatory cytokines interleukin-6 (IL-6), interleukin-10 (IL-10), and interleukin-1 beta (IL-1β).

**Methods:**

A cross-sectional study was conducted involving 100 participants categorized into four groups: IR only (n=30), T2DM without IR (DM-IR, n=20), T2DM with IR (DM+IR, n=20), and healthy controls (HC, n=30). mRNA levels of the cytokines in peripheral blood mononuclear cells (PBMCs) were quantified using qRT-PCR, and serum protein levels were assessed via ELISA.

**Results:**

Significant upregulation of IL-6 mRNA in PBMCs was observed in the DM+IR and DM-IR groups compared to controls and the IR only group, while serum IL-6 protein was paradoxically lower in DM+IR compared to DM-IR. Both IL-10 mRNA and serum protein levels were elevated in the DM-IR group, suggesting a compensatory anti-inflammatory response, but were downregulated in the DM+IR group. IL-1β showed a similar pattern, with increased mRNA and serum protein in DM-IR and decreased serum protein in DM+IR.

**Conclusion:**

The findings reveal distinct inflammatory cytokine profiles associated with IR and T2DM, highlighting discrepancies between mRNA and protein levels that suggest complex post-transcriptional and translational regulation. These results emphasize the necessity for careful interpretation of cytokine expression in the context of metabolic disorders.

## Introduction

The global prevalence of type 2 diabetes mellitus (T2DM) and insulin resistance (IR) has reached epidemic proportions, placing an immense burden on healthcare systems worldwide (WHO, 2024). These metabolic disorders are not solely defined by impaired glucose regulation but are increasingly recognized as chronic, low-grade inflammatory conditions (1). This persistent inflammatory state plays a central role in the development and progression of both IR and T2DM and contributes to the onset of microvascular and macrovascular complications (2). Moreover, experimental studies have demonstrated the direct impact of these conditions on the structural and functional integrity of pancreatic islets, underscoring the systemic nature of T2DM (3).

According to the International Diabetes Federation (IDF) Diabetes Atlas (2024), approximately 589 million adults globally are living with diabetes, the majority of whom have T2DM. The incidence of T2DM is rising rapidly, particularly in low- and middle-income countries, contributing to an escalating healthcare burden. In 2024, global diabetes-related healthcare expenditures reached an estimated USD 1 trillion. IR, a key precursor to T2DM, affects a substantial portion of the population and is associated with a range of comorbidities, including cardiovascular disease, non-alcoholic fatty liver disease, and polycystic ovary syndrome (4,5).

Cytokines such as interleukin-6 (IL-6), interleukin-10 (IL-10), and interleukin-1 beta (IL-1β) play critical roles in the complex interaction between inflammation and metabolic dysfunction. IL-6, a pleiotropic cytokine, has been shown to contribute to both insulin resistance and pancreatic β-cell dysfunction, serving as a molecular link between inflammation and metabolic dysregulation (6). IL-10, an anti-inflammatory cytokine, is believed to exert protective effects by counterbalancing pro-inflammatory signaling; however, its regulatory function may be compromised in chronic metabolic diseases (7). IL-1β, a potent pro-inflammatory cytokine, is involved in inflammasome activation and is considered a key mediator of inflammation in T2DM (8).

Although numerous studies have investigated the individual roles of these cytokines in IR and T2DM, the expression dynamics and circulating levels of IL-6, IL-10, and IL-1β in individuals with varying degrees of metabolic dysfunction remain incompletely understood. In particular, the differential effects of isolated IR, T2DM without IR, and the co-occurrence of both conditions on cytokine profiles warrant further investigation. Most existing studies have focused on either mRNA or protein levels alone, and few have clearly distinguished between the clinical subgroups of IR, T2DM without IR, and T2DM with IR (9).

This study addresses this critical gap by comprehensively analyzing both mRNA and protein levels of IL-6, IL-10, and IL-1β across well-defined metabolic groups. This dual-level approach offers a more nuanced understanding of the relationship between metabolic states and cytokine regulation. Furthermore, the discrepancy between cytokine mRNA expression in peripheral blood mononuclear cells (PBMCs) and corresponding protein concentrations in serum reflects the complexity of post-transcriptional and translational regulation. These layers of biological control can significantly influence cytokine bioavailability, emphasizing the importance of examining both gene expression and protein production (10).

Therefore, this study aimed to investigate the expression and circulating levels of IL-6, IL-10, and IL-1β in individuals with IR, T2DM without IR, T2DM with IR, and healthy controls. Using quantitative real-time PCR (qRT-PCR) and enzyme-linked immunosorbent assay (ELISA), we sought to clarify how these metabolic conditions shape the inflammatory cytokine landscape and to explore potential discordance between mRNA and protein levels. We hypothesized that the co-presence of IR and T2DM would produce a distinct cytokine profile, reflecting the synergistic impact of these disorders on inflammatory pathways. In addition, we aimed to generate insights into the regulatory mechanisms governing cytokine synthesis and release, which may inform the development of targeted therapeutic strategies.

## Materials and Methods

Study Participants and Group Allocation

This prospective cohort study was conducted at the Diabetes and Metabolic Disorders Department of Al-Forat Al-Awsat Hospital (Najaf, Iraq) between October 1, 2024, and January 22, 2025. A total of 100 participants were consecutively enrolled and stratified into four distinct groups based on established clinical and laboratory criteria for diabetes mellitus (DM) and insulin resistance (IR). Diagnostic criteria for DM followed the American Diabetes Association (ADA) guidelines, incorporating assessments such as glycated hemoglobin (HbA1c), fasting plasma glucose (FPG), oral glucose tolerance test (OGTT), and random plasma glucose (RPG).

The group classification was as follows:

- **Group 1: Insulin Resistance (IR) (n = 30)**
- Diagnosed with insulin resistance by a board-certified endocrinologist according to ADA criteria.
- **Group 2: Diabetes Mellitus with Insulin Resistance (DM + IR) (n = 20)**
- Diagnosed with both T2DM and IR based on ADA and WHO criteria.
- **Group 3: Diabetes Mellitus without Insulin Resistance (DM − IR) (n = 20)**
- Diagnosed with T2DM per ADA and WHO guidelines but did not meet IR criteria upon clinical and laboratory evaluation.
- **Group 4: Healthy Controls (HC) (n = 30)**
- Age- and sex-matched individuals with no personal history of DM or IR. Health status was confirmed via standard clinical assessments and laboratory investigations.

Sample Collection

Each participant provided two biological samples: one serum sample and one whole blood sample. Serum was immediately aliquoted and stored at −80°C. Peripheral blood mononuclear cells (PBMCs) were isolated from whole blood for downstream RNA extraction and cytokine analysis. Quantitative real-time PCR (qRT-PCR) was used to assess IL-6, IL-10, and IL-1β mRNA expression in PBMCs, while serum protein concentrations were quantified using enzyme-linked immunosorbent assay (ELISA).

Isolation of Peripheral Blood Mononuclear Cells (PBMCs)

PBMCs were isolated using Ficoll density gradient centrifugation per the manufacturer's protocol (Solar-Bio, Lot No. P4350), with minor modifications. Briefly, 2 mL of whole blood was diluted with 2 mL of sterile phosphate-buffered saline (PBS) in a 50 mL conical tube. In another 50 mL tube, 2 mL of Ficoll-Hypaque was layered beneath the diluted blood using a Pasteur pipette. Centrifugation was performed at 300 × g for 40 minutes at room temperature, brake off.

Four layers formed: plasma (top), PBMCs (interface), Ficoll-Hypaque, and red blood cells (bottom). The PBMC layer was carefully extracted and washed twice with PBS (centrifuged at 300 × g for 10 minutes per wash). The resulting pellet was resuspended in 1 mL of freezing medium (FSC medium with 5% dimethyl sulfoxide [DMSO]). Cells were counted using an automated cell counter (Bio-Rad) and adjusted to a final concentration of 2 × 10⁶ cells/mL. PBMC suspensions were cryopreserved in liquid nitrogen until analysis.

Gene Expression Analysis by qRT-PCR

PBMCs were processed to measure the expression of IL-6, IL-10, and IL-1β mRNA as potential biomarkers of IR and T2DM. Total RNA was extracted using the Solarbio Life Science RNA extraction kit, following the manufacturer’s instructions. Cells were centrifuged at 200 × g for 5 minutes, lysed, and RNA was purified via sequential washes using RPE and WT buffers. Purified RNA was eluted and stored at −20°C.

Complementary DNA (cDNA) was synthesized through reverse transcription. Quantitative PCR was performed on a Gene-line 7900HT Fast Real-Time PCR System using the Primer Design Precision 2× SYBR Green qPCR Master Mix. Validated primers targeting **GAPDH, IL-6, IL-10, and IL-1β** (Macrogen, South Korea) were used. Amplification proceeded for 40 cycles. Gene expression was analyzed using the comparative cycle threshold (Ct) method, normalized to **GAPDH** as the internal control (11).

All primers were custom-designed for this study, with sequences validated via NCBI BLAST (https://blast.ncbi.nlm.nih.gov/Blast.cgi).

**Table 1:** Primer Sequences and PCR Product Sizes for IL-6, IL-10, IL-1β, and GAPDH

Name	Human primers	PCR product
IL-6 F	CCACCGGGAACGAAAGAGAA	92
IL-6 R	GAGAAGGCAACTGGACCGAA	92
IL-10 F	CGAGATGCCTTCAGCAGAGT	55
IL-10 R	GGCAACCCAGGTAACCCTTA	55
IL-1β F	CCAAACCTCTTCGAGGCACA	86
IL-1β R	GGCTGCTTCAGACACTTGAG	86
GAPDH F	GTCGGAGTCAACGGATTTGG	165
GAPDH R	GACGGTGCCATGGAATTTGC	165

**Forward (F) and reverse (R) primer sequences used for quantitative real-time PCR (qRT-PCR) amplification of human IL-6, IL-10, IL-1β, and GAPDH genes. The expected PCR product sizes are listed in base pairs (bp).**

ELISA Preparation

Estimation of Human IL-6 Concentrations

Serum IL-6 levels were measured using a commercially available enzyme-linked immunosorbent assay (ELISA) kit (Solarbio, Beijing Solarbio Science & Technology Co., Ltd., catalog no. SEKH-0013), following the manufacturer’s instructions. After a brief centrifugation to remove particulate matter, IL-6 assays were initiated immediately.

A 1000 pg/mL stock standard was prepared by reconstituting the standard with 1.3 mL of standard sample diluent. Serial dilutions were performed by adding 250 µL of the 1000 pg/mL stock to 750 µL of diluent to create a 250 pg/mL solution. Additional serial dilutions were then performed to generate a standard curve.

For the assay, 100 µL of standard or sample was added to each well of the microtiter plate and incubated for 120 minutes at 37°C in sealed conditions. Wells were washed four times with 1× wash buffer. Then, 100 µL of biotin-conjugated anti-human IL-6 antibody working solution was added to each well and incubated for 60 minutes at 37°C, followed by four additional washes. Subsequently, 100 µL of HRP-avidin working solution was added and incubated for 30 minutes at 37°C. After five washes, 100 µL of TMB substrate was added, and the plate was incubated in the dark at 37°C for 5–15 minutes. The reaction was stopped by adding 50 µL of stop solution. Optical density was measured at 450 nm within 5 minutes using a microplate reader. IL-6 concentrations in unknown samples were interpolated from the standard curve.

Estimation of Human IL-10 Concentrations

Serum IL-10 levels were assessed using an ELISA kit (Solarbio, catalog no. SEKH-0018), according to the manufacturer’s protocol. After centrifugation to remove debris, samples were processed immediately.

A 1000 pg/mL stock standard was prepared by reconstituting with 1.0 mL of sample diluent. To prepare a 500 pg/mL standard, 500 µL of the stock was added to 500 µL of diluent, followed by serial dilutions to generate a full standard curve.

Each well received 100 µL of either standard or sample. Plates were incubated for 120 minutes at 37°C with gentle shaking (100 rpm) under sealed conditions. Wells were washed four times with 1× wash buffer. Then, 100 µL of a 1:100 streptavidin-HRP detector solution (combined with the diluted IL-10 antibody solution) was added and incubated for 60 minutes at 37°C with shaking. Wells were washed five times before 100 µL of TMB substrate was added. After incubation in the dark at 37°C for 5–30 minutes, the reaction was stopped with 50 µL of stop solution. Absorbance was read at 450 nm within 5 minutes, and IL-10 concentrations were determined via standard curve interpolation.

Estimation of Human IL-1β Concentrations

IL-1β concentrations in serum were determined using an ELISA kit (Solarbio, catalog no. SEKH-0002) per the manufacturer’s protocol. Following sample centrifugation, a 2000 pg/mL stock standard was reconstituted with 1.1 mL of sample diluent. A 1000 pg/mL standard was prepared by mixing 500 µL of the stock with 500 µL of diluent, followed by serial dilutions for the standard curve.

Each well received 100 µL of either standard or sample and was incubated for 120 minutes at 37°C with shaking (100 rpm). After four washes with 1× wash buffer, 100 µL of biotin-conjugated anti-human IL-1β antibody working solution was added and incubated for 60 minutes at 37°C with shaking. The plate was then washed four times before adding 100 µL of HRP-avidin solution and incubating for 30 minutes at 37°C with shaking. After five final washes, 100 µL of TMB substrate was added, and the reaction proceeded in the dark for 5–30 minutes. The reaction was stopped with 50 µL of stop solution. Optical density was read at 450 nm within 5 minutes. Concentrations were calculated by interpolation from the standard curve.

Statistical Analyses

All statistical analyses and graph generation were conducted using GraphPad Prism version 10. Comparisons among the experimental groups were performed using one-way analysis of variance (ANOVA), appropriate for evaluating differences across multiple groups. Post hoc tests, either Tukey’s or Bonferroni, depending on the comparison, were applied to determine specific group differences while correcting for multiple testing.

Data are presented as mean ± standard error of the mean (SEM). Prior to statistical testing, assumptions of normality and homogeneity of variance were confirmed, ensuring the suitability of parametric methods. Statistical significance was defined as *P* <0.05.

## Results

The changes in the IL-6 at mRNA and protein level in diabetes and insulin resistance cases

**Fig 1:**
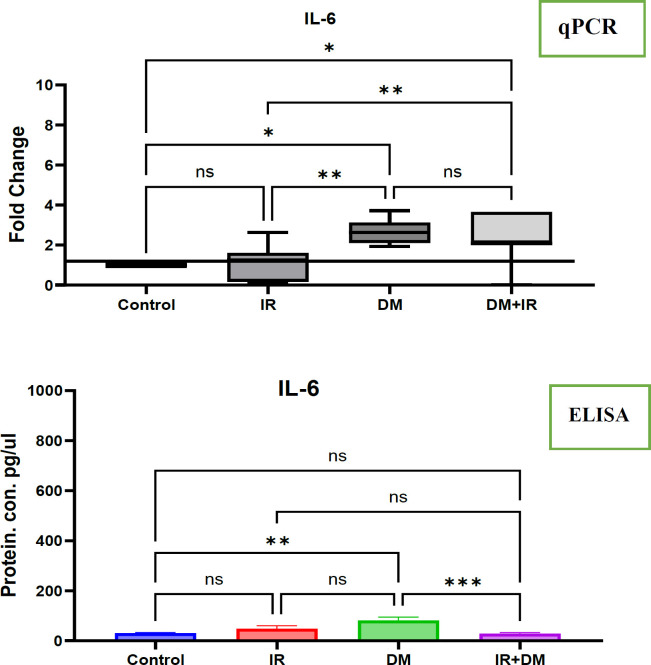
The IL-6 mRNA and protein expression has changed in response to diabetes mellitus and insulin resistance

This study investigated the role of interleukin-6 (IL-6), a pro-inflammatory cytokine, in the context of insulin resistance (IR) and type 2 diabetes mellitus (T2DM). We examined both gene expression (real-time PCR in PBMCs) and protein levels (ELISA in serum) of IL-6 in four groups: individuals with IR only, individuals with T2DM without IR (DM without IR), individuals with both T2DM and IR (DM with IR), and healthy controls. Our real-time PCR results demonstrated a significant upregulation of IL-6 mRNA in PBMCs from the DM with IR group compared to both healthy controls (p<0.1) and the IR only group (p<0.01). We also observed significantly increased IL-6 mRNA levels in the DM without IR group compared to both controls (p<0.1) and the IR group (p<0.01). Interestingly, no significant difference in IL-6 mRNA levels was found between the two diabetic groups. As shown in Fig 1, our ELISA results revealed significantly higher serum IL-6 protein levels in the DM without IR group compared to controls (p<0.01). Surprisingly, we did not observe significant differences in serum IL-6 protein levels between the DM with IR group and either the control or IR groups. We observed a significant decrease in serum IL-6 protein levels in the DM with IR group compared to the DM without IR group (p<0.001). 

**qPCR**: This figure illustrates the changes in IL-6 mRNA expression measured by qPCR in patients with insulin resistance and diabetes mellitus with and without insulin resistance compared with healthy cases. Expression of IL-6 in healthy and non-healthy cases was calculated using the 2-ΔΔCt method following estimation of the housekeeping gene GAPDH. IL-6 showed changes in PBMCs. The significance of differences was tested by one-way ANOVA , where * p<0.1, ** p<0.01, *** p<0.001, and **** p<0.0001 is significant, and ns is non-significant.

**The IL-6 protein **expression has been measured by Eliza in patients with insulin resistance and diabetes mellitus with and without insulin resistance compared with healthy cases. Expression of IL-6 in healthy cases and non-healthy cases was calculated using the standard curve method following an estimation of manufacturer information. **IL-6 **showed changes in serum. The significance of differences has been tested by one-way ANOVA, where **** p<0.0001 is significant, ** p<0.01 is significant and ns is non-significant.

The changes in the IL-10 at mRNA and protein level in diabetes and insulin resistance cases.

This study delved into the intricate role of interleukin-10 (IL-10), a crucial anti-inflammatory cytokine, in the context of diabetes mellitus and insulin resistance. The findings revealed a striking pattern in the DM-IR group. Compared to both the healthy controls and the individuals with insulin resistance alone, patients with diabetes but without insulin resistance exhibited a highly significant upregulation (p<0.0001) in both their IL-10 mRNA levels within PBMCs and the corresponding IL-10 protein levels circulating in their serum. This robust increase suggests a potential involvement of IL-10 in the specific context of diabetes when insulin sensitivity is maintained. Conversely, a different picture emerged in the DM+IR group. Despite showing an increase in IL-10 mRNA expression compared to the healthy controls (as detected by PCR), these individuals with both diabetes and insulin resistance demonstrated a significant downregulation (p<0.0001) in both IL-10 mRNA and serum protein levels when compared directly to the DM-IR group. This observation hints at a complex interplay between insulin resistance and IL-10 regulation in the diabetic state, potentially dampening the otherwise elevated IL-10 levels seen in diabetes without insulin resistance. Interestingly, the study found no significant alterations in IL-10 levels, neither at the mRNA nor the protein level, in individuals with insulin resistance alone when compared to the healthy control group. This suggests that insulin resistance per se might not be a primary driver of changes in systemic IL-10.

**Fig 2:**
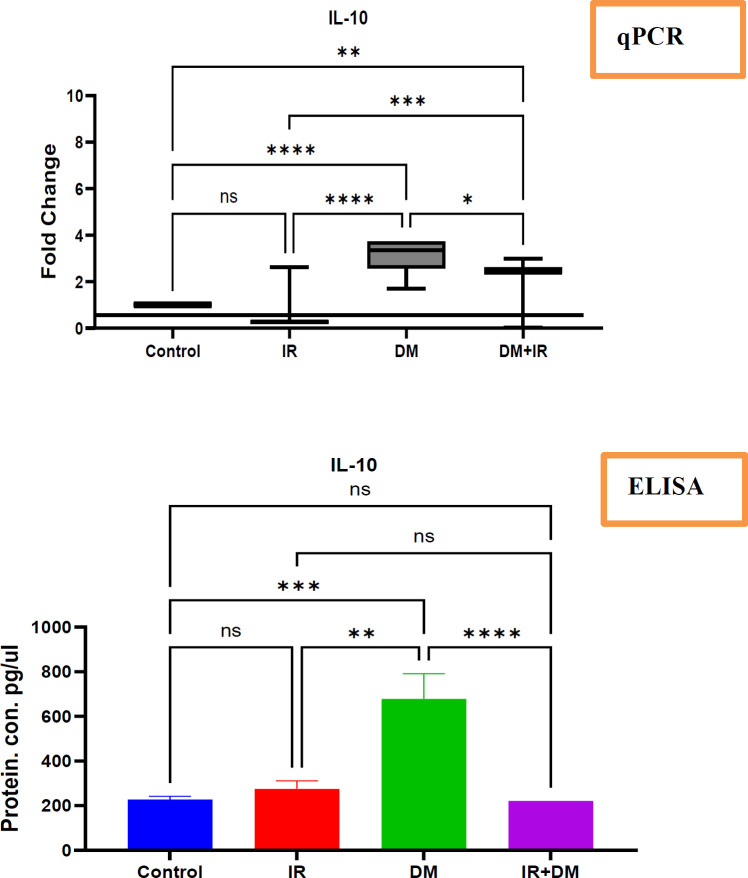
The IL-10 mRNA and protein expression has changed in response to diabetes mellitus and insulin resistance

**qPCR**: This figure illustrates the changes in IL-10 mRNA expression measured by qPCR in patients with insulin resistance and diabetes mellitus with and without insulin resistance compared with healthy cases. Expression of IL-10 in healthy and non-healthy cases was calculated using the 2-ΔΔCt method following estimation of the housekeeping gene GAPDH. IL-10 showed changes in PBMCs. The significance of differences was tested by one-way ANOVA , where * p<0.1, ** p<0.01, *** p<0.001, and **** p<0.0001 is significant, and ns is non-significant.

**The IL-10** protein expression has been measured by Elisa in patients with insulin resistance and diabetes mellitus with and without insulin resistance compared with healthy cases. Expression of IL-10 in healthy cases and non-healthy cases was calculated using the standard curve method following an estimation of manufacturer information. IL-10 showed changes in serum. The significance of differences has been tested by one-way ANOVA, where **** p<0.0001 is significant, ** p<0.01 is significant and ns is non-significant.

The changes in the IL-1β at mRNA and protein level in diabetes and insulin resistance cases

**Figure 3:**
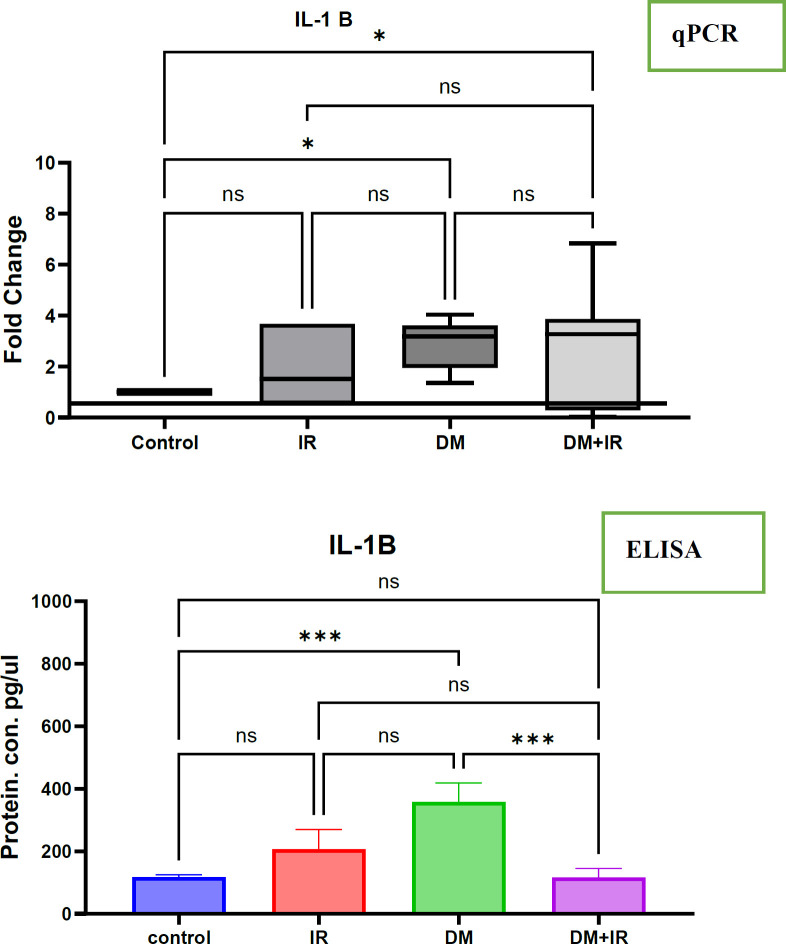
The IL-1β mRNA and protein expression has changed in response to diabetes mellitus and insulin resistance

The investigation explored the involvement of interleukin-1B (IL-1B) in diabetes mellitus and insulin resistance, revealing distinct patterns across the study groups. Notably, individuals with diabetes but without insulin resistance (DM-IR) displayed a significant increase (p<0.01) in both IL-1B mRNA levels in peripheral blood mononuclear cells (PBMCs) and circulating serum IL-1B protein levels when compared to both healthy controls and those with insulin resistance alone. This upregulation of IL-1B in the DM-IR group suggests a potential role for this cytokine in the pathophysiology of diabetes in the absence of insulin resistance (Figure 3).

In contrast, the group with both diabetes and insulin resistance (DM+IR) presented a different profile. While their IL-1B mRNA expression, as measured by PCR, showed no significant difference from the healthy controls, comparisons with the DM-IR group revealed non-significant changes in both IL-1B mRNA and serum protein levels. This suggests that the presence of insulin resistance alongside diabetes might modulate the IL-1B response observed in individuals with diabetes alone. Interestingly, the study found no significant alterations in IL-1B mRNA or protein levels in individuals with insulin resistance alone when compared to the healthy control group. This observation implies that insulin resistance itself might not be a primary factor influencing systemic IL-1B levels, highlighting the complex interplay between diabetes and insulin resistance in the context of inflammatory cytokine regulation.

**qPCR**: This figure illustrates the changes in IL-1β mRNA expression measured by qPCR in patients with insulin resistance and diabetes mellitus with and without insulin resistance compared with healthy cases. Expression of IL-1β in healthy and non-healthy cases was calculated using the 2-ΔΔCt method following estimation of the housekeeping gene GAPDH. IL-1β showed changes in PBMCs. The significance of differences was tested by one-way ANOVA , where * p<0.1, ** p<0.01, *** p<0.001, and **** p<0.0001 is significant, and ns is non-significant.

**The IL-1β **protein expression has been measured by Elisa in patients with insulin resistance and diabetes mellitus with and without insulin resistance compared with healthy cases. Expression of IL-1β in healthy cases and non-healthy cases was calculated using the standard curve method following an estimation of manufacturer information. IL-1β showed changes in serum. The significance of differences has been tested by one-way ANOVA, where **** p<0.0001 is significant, ** p<0.01 is significant and ns is non-significant.

## Discussion

The interplay between type 2 diabetes mellitus (T2DM) and insulin resistance (IR) significantly impacts the inflammatory milieu, as evidenced by alterations in interleukin (IL)-6, IL-10, and IL-1β expression and circulating levels. Our findings reveal a synergistic upregulation of IL-6 mRNA in PBMCs from individuals with both DM and IR compared to healthy controls (p<0.1) and those with IR alone (p<0.01), underscoring an amplified inflammatory state in the combined condition, consistent with prior research (12, 13). Notably, T2DM alone also led to elevated IL-6 mRNA in PBMCs (p<0.1 vs. controls, p<0.01 vs. IR), aligning with studies demonstrating that hyperglycemia can independently trigger IL-6 production (14, 15). The absence of a significant difference in IL-6 mRNA between the two diabetic groups suggests a potential ceiling effect or distinct regulatory mechanisms (16).

However, serum IL-6 protein levels presented a more complex picture. While T2DM without IR showed significantly higher serum IL-6 compared to controls (p<0.01), corroborating the mRNA findings and the role of hyperglycemia (17, 18), no such increase was observed in the DM with IR group. This discrepancy between mRNA and protein levels could be attributed to post-transcriptional regulation (19) or differential contributions from various cell types (20). Intriguingly, serum IL-6 protein was significantly lower in the DM with IR group compared to the DM without IR group (p<0.001), potentially indicating sequestration in tissues like adipose or muscle (21) or altered production/clearance dynamics in the combined condition (22), warranting further investigation.

Regarding the anti-inflammatory cytokine IL-10, we observed a disparity between PBMC mRNA and serum protein levels, highlighting the importance of post-transcriptional control (10).The significant upregulation of both IL-10 mRNA and serum protein in the DM without IR group compared to controls and the IR group suggests a compensatory anti-inflammatory response potentially mediated by increased regulatory T cells attempting to counteract hyperglycemia-induced inflammation (23). Conversely, the downregulation of IL-10 mRNA and serum protein in the DM with IR group compared to the DM without IR group, despite increased mRNA compared to controls, indicates a potential failure of this counter-regulatory mechanism in the presence of IR, possibly due to IR-associated chronic inflammation impairing IL-10 production or stability (24). The lack of significant IL-10 changes in the IR group alone suggests that hyperglycemia may be the primary driver of IL-10 alterations in this context. The differential expression between PBMCs and serum may reflect varied rates of cytokine release (25) (26).

Similar to IL-10, IL-1β displayed a dissociation between mRNA and protein levels, underscoring the multifaceted regulation of cytokine production, including post-transcriptional modifications and the requirement for post-translational processing for biological activity (27, 28). The significant upregulation of both IL-1β mRNA and serum protein in the DM without IR group points to heightened inflammatory activity driven by hyperglycemia, potentially through activation of the NLRP3 inflammasome (29). The observed downregulation of serum IL-1β in the DM with IR group compared to the DM without IR group, despite a similar trend in mRNA upregulation, suggests a complex modulation of IL-1β release or activity in the presence of IR (30). The interplay of inflammatory mediators in the combined condition might lead to a distinct cytokine profile. The absence of significant IL-1β changes in the IR group alone suggests that hyperglycemia is a key inducer of IL-1β in this setting, although further research with detailed IR characterization is warranted. The discrepancy between PBMC and serum levels may indicate regulated protein release. These findings align with established roles of IL-1β in T2DM and IR pathogenesis (31) (32) and the differences between the diabetic groups highlight how the presence of IR modifies the inflammatory landscape.

## Conclusion

This study reveals a distinct and intricate modulation of IL-6, IL-10, and IL-1β in insulin resistance and type 2 diabetes, evidenced by a notable dissociation between mRNA and serum protein levels. The divergent inflammatory profiles observed between the DM-IR (upregulated IL-6 and IL-1β, robust IL-10) and DM+IR (downregulated IL-6 and IL-1β, impaired IL-10 response) groups underscore the significant impact of insulin resistance on the diabetic inflammatory milieu. Furthermore, the discrepancies between cellular and systemic cytokine levels highlight the complexity of cytokine biology beyond transcriptional regulation. These findings necessitate further longitudinal studies with larger cohorts to validate these observations and elucidate the underlying post-transcriptional mechanisms and the involvement of diverse cell types in shaping the inflammatory landscape of these metabolic disorders, potentially informing targeted therapeutic interventions.

## Limitation 

The current study, while insightful, presents several limitations that should be acknowledged to enhance its rigor for high-quality publication. Firstly, the manuscript would benefit from explicitly stating the sample size of each study group and detailing the inclusion/exclusion criteria, particularly the definition of insulin resistance and the characteristics of the T2DM participants. Secondly, the reliance on PBMCs as a surrogate for target tissues involved in metabolic dysfunction warrants caution, as these may not fully reflect local inflammatory processes. Future studies should aim to investigate cytokine expression in tissues like adipose, muscle, and pancreas. Furthermore, the cross-sectional design of this study limits the ability to establish temporal relationships between cytokine changes and disease progression. Longitudinal studies are needed to track these dynamics. While potential mechanisms are discussed, the study does not directly explore them. Future research should employ mechanistic approaches to elucidate the underlying regulatory pathways. Additionally, the focus on a limited panel of cytokines (IL-6, IL-10, IL-1β) provides an incomplete picture of the inflammatory milieu. Expanding the analysis to include other relevant markers would offer a more comprehensive understanding. Finally, the interpretation of trends with p<0.1 requires caution due to the lack of statistical significance, and unaddressed confounding factors, such as medication use and comorbidities, could have influenced the results. Future studies should aim to control for these variables to strengthen the validity of the findings.

## Data Availability

There is no additional data separate from available in cited references.
